# Fitness Costs of Tigecycline Resistance in *Acinetobacter baumannii* and the Resistance Mechanism Revealed by a Transposon Mutation Library

**DOI:** 10.3390/antibiotics11101379

**Published:** 2022-10-09

**Authors:** Ping Wang, Hongou Wang, Cunwei Liu, Chengjie Feng, Qinghui Lu, Qinghua Zou

**Affiliations:** 1 Department of Microbiology, School of Basic Medical Sciences, Peking University Health Science Center, Beijing 100191, China; 2 Peking University People’s Hospital, Beijing 100044, China

**Keywords:** *Acinetobacter baumannii*, tigecycline, mechanism of resistance, fitness costs, transposon mutation library

## Abstract

*Acinetobacter baumannii* is one of the main pathogens causing nosocomial and community-acquired infections. Tigecycline is an important antibiotic for the treatment of multidrug-resistant *A. baumannii* infections, but strains resistant to tigecycline have also emerged. There are still many unclear questions concerning the mechanism of tigecycline resistance in *A. baumannii*. In this study, tigecycline-susceptible and tigecycline-intermediate strains were gradually cultured with sub-minimum inhibitory concentrations of tigecycline to select for tigecycline-resistant mutants, and a tigecycline-resistant strain was cultured under 42 °C to select for tigecycline-susceptible mutants. We found that the acquisition of tigecycline resistance affected the susceptibility of the strains to other antibiotics. Resistance to ampicillin–sulbactam is negatively correlated with tigecycline resistance. The strains will experience fitness costs along with the acquisition of tigecycline resistance. Tigecycline resistance in the strains was not related to 16S rRNA target variation or outer membrane integrity alteration. By constructing a transposon mutation library, we found that transposon insertion of the *adeL* gene reduced the sensitivity of *A. baumannii* to tigecycline. This study provides important clues for understanding the mechanism of tigecycline resistance in *A. baumannii*.

## 1. Introduction

*Acinetobacter baumannii* is one of the most ubiquitous opportunistic pathogens that predominantly causes nosocomial infections in ill patients, especially in intensive care units (ICUs) [1]. *A. baumannii* infection can lead to ventilator-associated pneumonia and bacteremia in critically ill patients, as well as meningitis, peritonitis, endocarditis, and skin infections [2,3]. Due to biofilm formation, drug resistance, desiccation tolerance, etc., *A. baumannii* exhibits strong environmental adaptability and transmissibility [4,5,6,7]. In addition, the prevalence of multidrug-resistant *A. baumannii* strains is increasing yearly. Crude mortality rates for *A. baumannii* infection ranged from 8% to 85% in susceptible populations and varied with patient status [8,9,10,11]. For instance, MDR *A. baumannii* infection occurring in the ICU had a mortality of 84.3% [11], which was much higher than that in normally sterile sites (41.3%) [9].

*A. baumannii* has also been confronted with serious drug resistance. Multidrug-resistant (MDR), extensively drug-resistant (XDR), and pandrug-resistant (PDR) *A. baumannii* have been widely found [12,13]. Their complex drug-resistant mechanisms and easy acquisition of new exogenous genes make most clinical isolates resistant to multiple antibiotics, such as β-lactams, fluoroquinolones, tetracyclines, and aminoglycosides [1,14]. Lob et al. investigated 2337 *A. baumannii* isolated from intra-abdominal infections and urinary tract infections from 2011 to 2014 at 453 hospital sites in 48 countries and revealed that the MDR rate was 47% to 93%, while the highest MDR rate was observed in ICUs in Europe and the Middle East [15]. It was also reported that 44% of clinically isolated strains were MDR among 18,741 *A. baumannii* collected from 2004 to 2014, of which 95% were resistant to ceftriaxone and nearly 90% were resistant to piperacillin–tazobactam, ceftazidime, levofloxacin, and meropenem [16]. The contradiction between the ever-growing prevalence of MDR and XDR and the inadequate development of antibiotics has made *A. baumannii* infection a public health crisis and a major difficulty in clinical treatment [17].

*A. baumannii* possesses high genome plasticity. It can easily obtain a multidrug-resistant phenotype by acquiring exogenous resistance elements through horizontal gene transfer [18]. It has been proven that the susceptibility of *A. baumannii* to polymyxin and tigecycline remains at relatively high levels [16,19]. In clinical treatment, polymyxin antibiotics are now considered to be the drug of choice for treating infections caused by carbapenem-resistant strains [20]. Due to the high adaptability of the strains, persistent or recurrent infection caused by drug-resistant bacteria often occurs during treatment [21]. Along with the high risk of nephrotoxicity (38.7%) caused by polymyxin B, tigecycline might become the last line of defense against *A. baumannii* [22,23]. However, with the rapid evolution of resistant strains, tigecycline-resistant strains have also emerged [24].

Increasing amounts of tigecycline-resistant strains have been reported in recent years, yet the mechanism of tigecycline resistance remains unclear [25]. Tigecycline is a derivative of minocycline, which inhibits protein synthesis by binding to the 30S ribosomal subunit, thereby blocking the interaction of aminoacyl-tRNA with the site of the ribosome [25]. Numerous studies have revealed that flavin-dependent monooxygenase (TetX) might be associated with tigecycline resistance and could be spread through plasmids [26,27]. The resistance of *A. baumannii* to tigecycline is also related to overexpression of the efflux pump, especially the resistance-nodulation-cell division (RND) superfamily efflux pump. Foong et al. stated that the tetracycline transporter (TetA) and RND-type transporters (AdeABC and AdeIJK) were indispensable for *A. baumannii* gaining tigecycline resistance though drug efflux [28]. In addition, previous studies elucidated that the deletion of the S-adenosyl-L-methionine-dependent methyltransferase encoding gene *trm* led to distinctly enhanced tigecycline resistance via an efflux pump-independent mechanism [29,30]. The above factors could only be identified in some drug-resistant bacteria; therefore, other possible resistance mechanisms need to be elucidated.

Transposon-insertion sequencing (TIS) was adopted in this study and our results indicated a close causality link between *adeL* expression and tigecycline resistance in *A. baumannii*. We simultaneously found that biofilm formation ability affected the susceptibility of *A. baumannii* to tigecycline. Our study highlights the application of TIS for drug-resistance gene screening and offers insights for antibiotic development.

## 2. Results

### 2.1. Selection of Tigecycline-Resistant or Tigecycline-Susceptible A. baumannii

Three tigecycline-intermediate strains, A8S, A9S, and A11S, and three tigecycline-susceptible strains, A152S, A158S, and 17978S, were incubated with successive sub-MICs of tigecycline and were successfully induced into strains with tigecycline-resistant phenotype A8R, A9R, A11R, A152R, A158R, and 17978R. The MICs for tigecycline of the strains increased from 0.5~4 to 64~128 μg/mL after induction (Table S1). Meanwhile, we induced tigecycline-resistant strain A54R into tigecycline-susceptible strain A54S by subculturing it for 18 generations (days) at 42 °C. The MIC of A54R was reduced from 8 μg/mL to 1 μg/mL after induction. All induced strains were sub-cultured for 15 generations (days) under non-tigecycline conditions and the tigecycline MICs remained constant after passaging (Table S1), suggesting that tigecycline resistance after induction is stable. It is worth noting that the induced tigecycline-susceptible strain A54S could recover its resistance to tigecycline. By comparing the growth of A54S on an LB solid medium containing 8 μg/mL tigecycline and an LB solid medium without any antibiotics, the recovery rate was approximately 0.0015‰ ± 0.0007‰.

### 2.2. The Acquisition/Loss of Tigecycline Resistance Affected Susceptibility to Other Antibiotics

**Figure 1:**
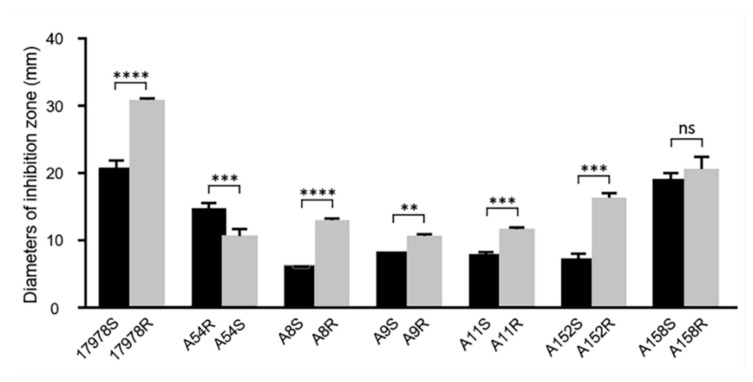
Diameter of the inhibition zone with the ampicillin/sulbactam Kirby–Bauer paper before and after induction. Data are presented as the mean ± SD; ns = not statistically significant, ** *p* < 0.01, *** *p* < 0.001, **** *p* < 0.0001.

It is unclear whether the acquisition/loss of tigecycline resistance can affect the susceptibility of the strains to other antibiotics. We tested the susceptibility of the strains to the other 15 antibiotics and found that susceptibility to these antibiotics varied after sub-MIC tigecycline induction (Table 1). In 17978R, minocycline resistance increased with the acquisition of tigecycline resistance, while piperacillin resistance decreased. The ampicillin–sulbactam resistance decreased in A8R and the resistance to ampicillin–sulbactam, ceftazidime, gentamicin, and cotrimoxazole decreased after A152R acquired tigecycline resistance. A158R acquired increased resistance to cefepime, gentamicin, and ciprofloxacin after tigecycline-resistance acquisition. Interestingly, we found that for six tigecycline-susceptible strains (17978S, A8S, A9S, A11S, A152S, and A158S), when they acquired tigecycline resistance, susceptibility to ampicillin–sulbactam increased, as reflected by the increase in the diameter of the inhibition zone of the antibiotic-susceptible paper (Figure 1). While A54R was inherently resistant to tigecycline, reduced susceptibility to ampicillin–sulbactam was observed after being induced into a tigecycline-susceptible strain (Figure 1).

Since the Kirby–Bauer method is a qualitative method, we used the broth dilution method to confirm the antibiotic susceptibility of the strains to AMS again. Except for 17978R and A158R, the MICs of the other induced tigecycline-resistant strains to AMS were all lower than those of the corresponding tigecycline-susceptible strains, which was consistent with the results of the Kirby–Bauer method (Table 2). To further explore whether ampicillin or sulbactam exerts this antibacterial activity in AMS, we tested the susceptibility of the strains to ampicillin and sulbactam and found that the MICs of the strains to ampicillin before and after induction showed no significant alteration. In contrast, the MICs to sulbactam of the resistant strains were 2~16-fold lower than those of the susceptible strains, except 17,978 S/R and A54 R/S (Table 2), suggesting that sulbactam played key roles in antibacterial activity. These results suggest that ampicillin–sulbactam may have a potential use in the treatment of tigecycline-resistant bacteria. We then detected the effect of the combination of ampicillin–sulbactam and tigecycline on the tigecycline-resistant strains A8R, A9R, A11R, A152R, and A158R using the checkerboard method. As expected, the combined use of the antibiotics had an additive effect on some strains (A8R and A9R) with FIC values of 1 and 0.625, respectively.

### 2.3. Acquisition of Tigecycline Resistance Elicited Fitness Costs in A. baumannii

**Figure 2:**
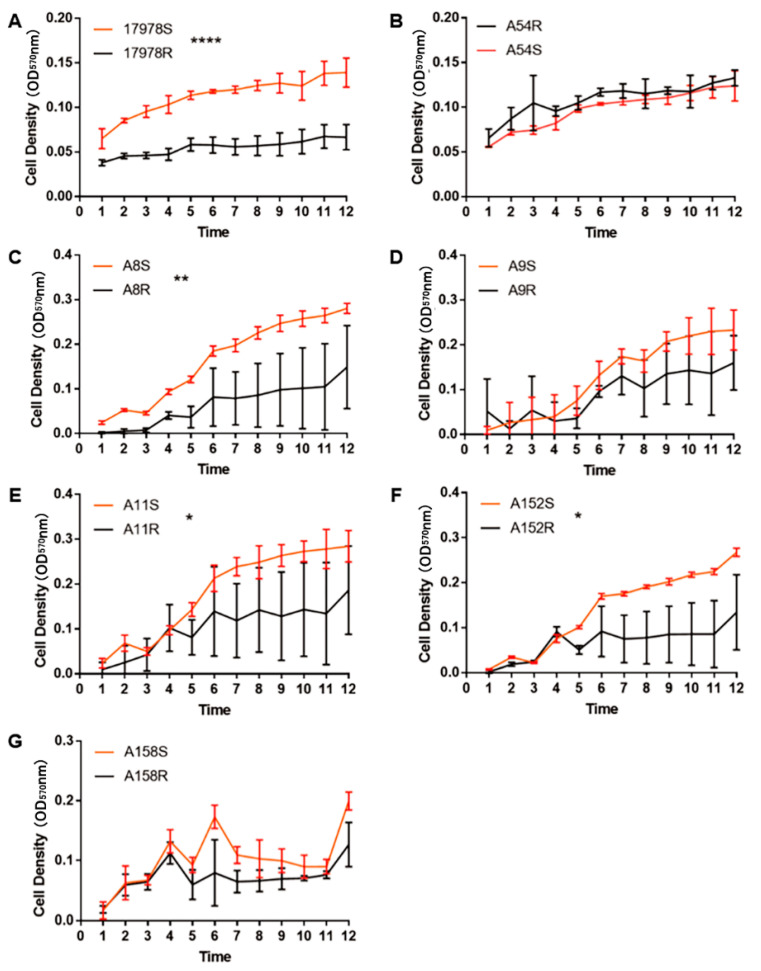
Growth curves of different strains of *A. baumannii* before and after induction of tigecycline resistance or susceptibility in M9 medium (**A**–**G**). Data are presented as the mean ± SD. * *p* < 0.05, ** *p* < 0.01, **** *p* < 0.0001.

**Figure 3:**
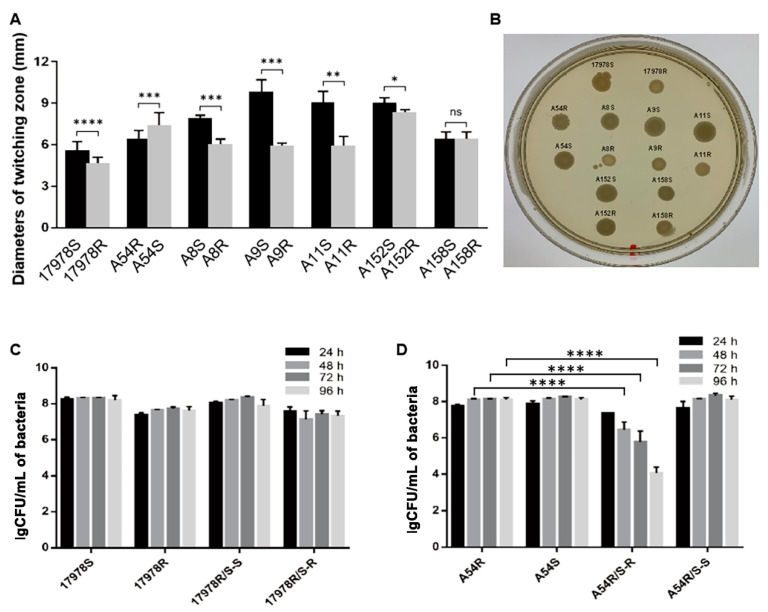
The mobility and competitive ability of *A. baumannii* before and after induction of tigecycline resistance or susceptibility. (**A**) Diameters of the twitching zone of different strains (mm). (**B**) Visualization of twitching zones of different strains. (**C**,**D**) Numbers of bacterial colonies in the individual cultured and cocultured competitiveness states. 17978S, 17978R, A54R, and A54S are the bacteria that were cultured alone, and R/S-R, S/R-R, and R/S-S, S/R-S represent the resistant and susceptible strains in cocultured states, respectively. Data are presented as the mean ± SD. * *p* < 0.05, ** *p* < 0.01, *** *p* < 0.001, **** *p* < 0.0001.

When bacteria develop antibiotic resistance, they may also suffer from fitness costs, including the inhibition of growth ability, competitiveness, and virulence [31]. In the 17978R, A8R, A11R, and A152R strains, the growth ability in M9 medium was markedly decreased after tigecycline resistance was obtained and downward trends were also observed in A9R and A158R (Figure 2). However, there was no difference in the growth ability of all strains before and after induction in LB (Figure S1). These data implied that tigecycline-resistant strains induced by sub-MIC tigecycline exhibited significant fitness costs of growth defects in a low-nutrient medium. Although *A. baumannii* lacks flagella, it can actually spread on the surface of a semisolid medium, which is called twitching motility [32]. The motility of 17978R, A8R, A9R, A11R, and A152R was drastically suppressed after acquisition of tigecycline resistance, while the motility of A54R was significantly enhanced after induction into tigecycline-susceptible strain A54S (Figure 3A,B), suggesting that tigecycline-resistant strains have motility ability costs. We further investigated the competitiveness of the strains using 17978 S/R and A54 R/S as representative, since they were induced in two different ways. As shown in Figure 3C,D, the ratio of tigecycline-resistant and tigecycline-susceptible bacteria did not change significantly after 17978 S/R cocultivation. While the number of A54R strains drastically decreased after A54 R/S cocultivation, its number gradually decreased with increasing coculturing time, suggesting that the competitiveness cost in A54R might be strain specific.

### 2.4. Biofilm Formation Ability Was Related to Tigecycline Resistance in A. baumannii

**Figure 4:**
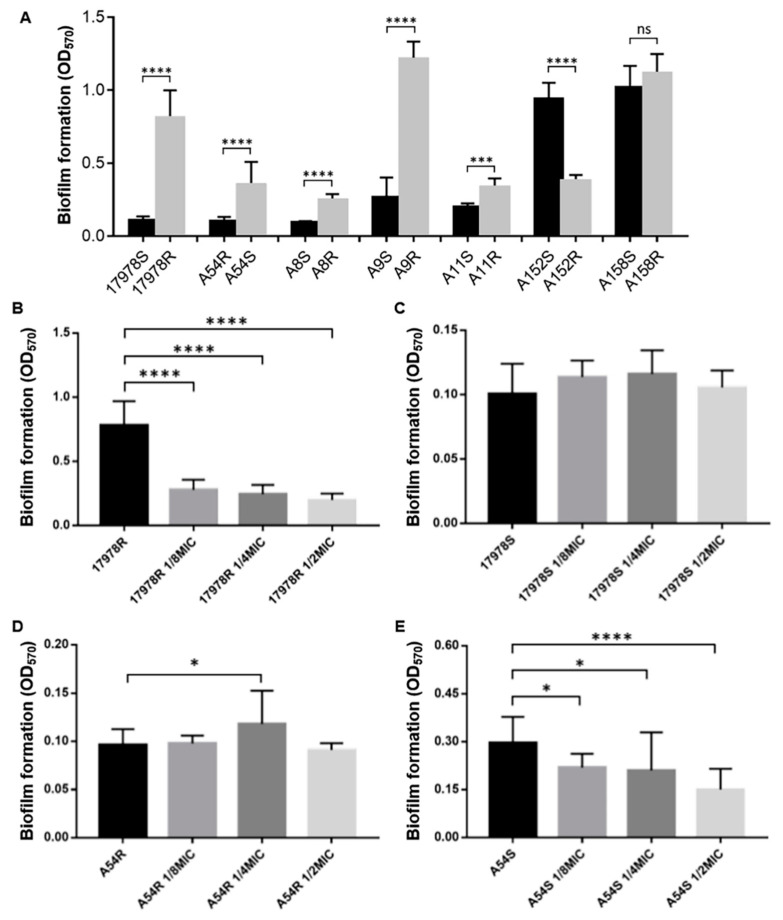
Biofilm formation ability. (**A**) Biofilm formation ability of the strains before and after induction of tigecycline resistance or susceptibility. (**B**–**E**) Biofilm formation ability of strains under sub-MICs of tigecycline. (**B**–**E**) show the biofilm formation ability of 17978R, 17978S, A54R, and A54S cultured without tigecycline at 1/8 MIC, 1/4 MIC, and 1/2 MIC. Formability is described by the OD570 value detected. Data are presented as the mean ± SD. * *p* < 0.05, *** *p* < 0.001, **** *p* < 0.0001.

Although the relationship between biofilms and bacterial resistance is still controversial, researchers generally believe that biofilms may be involved in drug resistance in *A. baumannii*. We next measured the biofilm formation ability of the strains and found inconsistent results before and after induction (Figure 4A). The biofilm formation ability of 17978R, A8R, A9R, and A11R was significantly enhanced after the acquisition of tigecycline resistance, yet this phenotype in A152R was the opposite. There was no significant difference between A158 S/R, but there was a trend of biofilm formation enhancement. Notably, the biofilm formation ability of A54R was enhanced after induction into the tigecycline-susceptible strain A54S.

To examine the correlation between the biofilm formation and tigecycline resistance of these strains, we subsequently determined the biofilm formation ability of 17978R/S and A54R/S under sub-MICs of tigecycline treatment. Neither of the doses of sub-MICs displayed a significant difference in 17978S, although there was a slight upward trend in 1/8 MIC and 1/4 MIC, while the biofilm formation abilities were dramatically repressed in the presence of sub-MICs of tigecycline in tigecycline-resistant strain 17978R (Figure 4B,C). Conversely, the biofilm formation ability in tigecycline-resistant strain A54R was upregulated after 1/4 MIC tigecycline incubation, while it decreased with increasing tigecycline concentrations in the corresponding tigecycline-susceptible strain A54S (Figure 4D,E). Additionally, the minimum biofilm clearance concentration was explored to clarify the effects of biofilms on tigecycline resistance. We found that the minimum biofilm clearance concentrations of 17978S, 17978R, A54R, and A54S were 32, 1024, 512, and 128 μg/mL, respectively. Taken together, these data indicate that higher tigecycline resistance emerged after biofilm formation than in the respective bacterioplankton states.

### 2.5. Tigecycline Resistance in A. baumannii Was Not Related to 16S rRNA Target Variation or Outer Membrane Integrity Alteration

**Figure 5:**
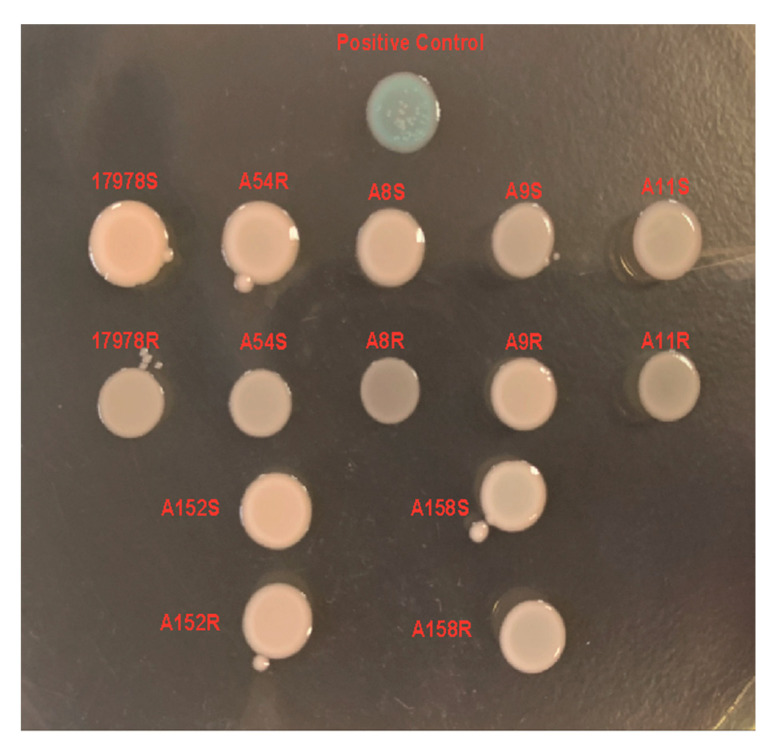
Cell membrane damage detected by XP. Bacteria with cell membrane damage formed blue clones. The positive control was the A54R strain treated with SDS.

The target of tigecycline is the bacterial ribosomal 30S ribosomal subunit and mutations in the tigecycline target located in this subunit were sufficient to endow *Klebsiella pneumonia* with tigecycline resistance [33,34]. It was also verified that 16S rRNA exists as an important component in the 30S ribosomal subunit and acts as the binding site for tigecycline [35]. We, thus, compared the sequences of 16S rRNA in the induced strains with their original strains. As shown in Figure S2, there was no site mutation before and after induction of each strain. Previous studies have reported that tigecycline resistance might be related to the permeability of the cell membrane [36]; hence, we further detected the cell membrane permeability of each strain with XP, which could react with alkaline phosphatase and form a blue precipitate [37]. Compared with the positive control (SDS-treated strain), outer membrane impairments were not observed in either sub-MIC-induced tigecycline-resistant strains or high-temperature-induced tigecycline-susceptible strains (Figure 5). Taken together, sub-MIC-induced tigecycline resistance barely depended on the 16S rRNA target variation or outer membrane integrity destruction.

### 2.6. The Deprivation of adeL Triggered Tigecycline Resistance in A. baumannii

**Figure 6:**
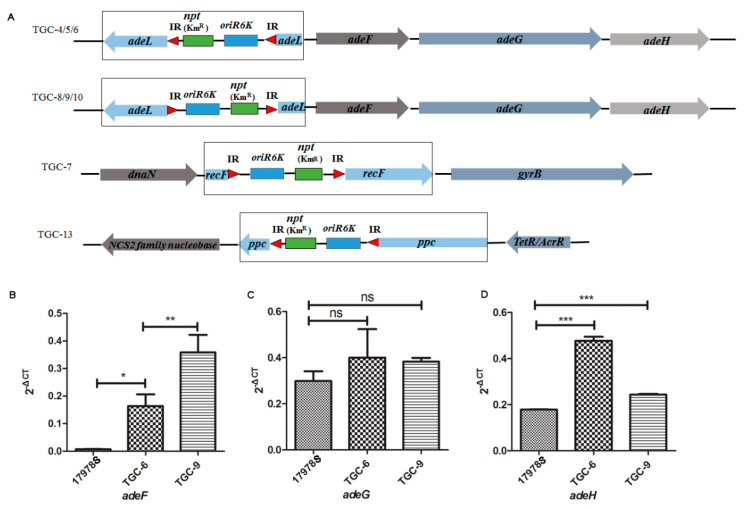
Transposon insertions in the mutants (**A**) and transcription level of *adeF* (**B**), *adeG* (**C**), and *adeH* (**D**) detected by qRT-PCR. * *p* < 0.05, ** *p* < 0.01, *** *p* < 0.001.

To better understand the underlying mechanisms by which sub-MICs induced tigecycline resistance in *A. baumannii*, TIS was employed to identify the key regulatory factors in this process. In this study, 17978S served as the recipient bacteria and the Tn5 transposon was randomly inserted into its genome by a conjugative transfer with the pRL27 plasmid [38]. We created a random mutation library containing 4500 mutant strains; 16 of them exhibited an obvious sub-MIC tigecycline-induced resistance phenotype, which was named TGC-1 to TGC-16. Among these mutants, TGC-1 to TGC-14 were successfully inserted with transposon sequences. The insertion site analysis revealed that in the mutant TGC-4/5/6/8/9/10, Tn5 was inserted into the *adeL* gene (Figure 6A). As a multidrug efflux transcriptional repressor, AdeL regulates the RND-type efflux system through the inhibition of the efflux system AdeFGH and prevents the efflux of antibiotics, which further restrains the generation of bacterial drug resistance [39]. Nevertheless, the directions of inserted Tn5 were different even when inserted in the same gene: TGC-4/5/6 were forward orientations and TGC-8/9/10 were reverse orientations (Figure 6A).

The disruption of *adeL* could be detected in 42.8% (6/14) of the transposon mutants, suggesting that it might play a pivotal role in tigecycline resistance in *A. baumannii*. Meanwhile, other mutant genes that indirectly correlated with the resistance phenotype were also observed. For example, Tn5 was inserted into *ppc* (a phosphoenolpyruvate carboxylase active protein encoding gene) [40] in TGC-13 (Figure 6A), while it was inserted into *recF* (encoding the DNA replication/repair protein, RecF) [41] in TGC-7 (Figure 6A). To further test the regulatory effects of *adeL* on tigecycline resistance in *A. baumannii*, we selected the transposon insertion mutant TGC-6 to rescue *adeL* by constructing the gene complementary strain with pTrc99A and found that the complement expression of *adeL* reduced the tigecycline resistance of the strain from 4 ng/μL to 2 ng/μL. We further tested the transcription levels of *adeF*, *adeG,* and *adeH* and found that the transcription levels of *adeF* and *adeH* were significantly increased in the insertion mutant TGC-6 and TGC-9 compared with their parent stain 17978S (Figure 6B). These findings indicate that AdeL negatively regulates the tigecycline resistance of *A. baumannii*.

## 3. Discussion

Although tigecycline is regarded as the last resort for treating MDR and XDR *A. baumannii* infections, the tigecycline resistance rate has risen in recent years [23,42,43,44]. However, the underlying mechanism is still not well understood. ATCC 17978 is a representative bacterial model that is commonly used in grouping for pathogenic and drug-resistance mechanisms. However, it is not indicative of characterizing *A. baumannii* due to the relatively weaker drug resistance and toxicity than that in clinical isolates [4,45]. The probability of drug-resistant mutations might be elevated under long-period sub-MIC antibiotic stresses caused by the presence of trace concentrations of antibiotics in the environment, uneven distributions of anti-infective drugs in host organs, and the long-term and non-standard use of antibiotics [46,47,48]. Therefore, our research implemented a sub-MIC induction strategy on tigecycline-susceptible and -intermediate strains and successfully obtained tigecycline-resistant strains to explore the key regulators in the development of bacteria resistance.

It has been comprehensively shown that most antibiotic resistance is involved in fitness costs and reduction in antibiotic content is beneficial to the growth of susceptible strains [31]. Compared with the corresponding tigecycline-susceptible or tigecycline-intermediate strains, we observed significant inhibition of the growth ability of sub-MIC-induced tigecycline-resistant strains in M9 medium, instead of LB broth. We hypothesized that growth restrictions might exist as a compensatory mechanism of tigecycline resistance, which transforms respiratory to glucose fermentative metabolism upon efflux pump overexpression [49]. At the same time, A54R showed a competitive cost when cocultured with A54S. In addition, the twitching motility of *A. baumannii* is related to biofilm formation and colonization in the bacteria host. Twitching motility is a flagella-independent behavior that is mediated by type IV pili and is conducive to bacterial survival under stressed circumstances [50,51]. Here, we revealed the marked suppression of motility in tigecycline-resistant strains, whether acquired resistance induced by sub-MICs or original resistance isolated from clinical cases. Our data were consistent with previous reports that various antibiotic resistances had the capability to elicit fitness costs in different bacterial strains [52,53,54].

Sulbactam is widely used as a stand-alone antibacterial agent for treating multidrug-resistant *A. baumannii* infection [14]. However, sulbactam-resistant strains are severely increasing, making the treatment less effective. Previous studies showed that the combination of high-dose sulbactam with other antibiotics, such as levofloxacin and tigecycline, achieved the highest antibacterial efficacy [55]. Ampicillin–sulbactam (ampicillin/sulbactam, AMS) is also an effective combination for the treatment of nosocomial *A. baumannii* [56,57] and the combination of tigecycline–sulbactam may also have a synergistic effect [58]. Assimakopoulos et al. found that high doses of tigecycline, AMS, and polymyxin could effectively treat ventilator-associated pneumonia caused by highly tigecycline-resistant strains [59]. Since sulbactam is a beta-lactamase inhibitor, it is reasonable to assume that there will be a better effect when combined with ampicillin. As can be seen in our study, the A8R strain was more sensitive to AMS than sulbactam alone. By testing the AMS resistance of the seven pairs of strains before and after tigecycline-resistant induction, we found that although they were not totally susceptible to AMS, the MIC to AMS of the tigecycline-resistant strains was 2~16-fold lower than that of the susceptible strains. The effect of the combination of AMS and tigecycline is consistent with other studies [60]. There is a synergistic effect when they are used in combination. The mechanism still needs further study, but AMS may be used as an important candidate to treat tigecycline-resistant strains to a certain extent.

Additionally, biofilm formation was also illustrated to enhance resistance to antimicrobial chemicals [61]. However, there is no consistent conclusion about the relationship between biofilm formation ability and tigecycline resistance in this study. Four of the seven pairs of strains used in this study showed strong biofilm formation ability in drug-resistant strains, while in the other pairs, the resistant strain also tended to be stronger. Unexpectedly, our findings failed to expound a consistent conclusion about the relationship between biofilm formation ability and tigecycline resistance.

Consistent with a previous study [62], the tigecycline resistance of biofilm strains in this study was much higher than that of planktonic bacteria alone, while the effects of the sub-MICs of tigecycline on biofilms varied among strains. On the one hand, the biofilm formation ability of the 17978R and A54S strains was significantly suppressed under sub-MICs of the tigecycline treatment, which was in accordance with some studies [62,63]. On the other hand, the results presented by Lin et al. were the opposite, in which the formation of biofilms showed no observational reduction at low concentrations of tigecycline and even an increased level at high concentrations [64]. Analogously, our data found that the sub-MICs of tigecycline resulted in an increasing trend of biofilm formation in 17978S, although there was a significant difference, while the biofilm formation ability of A54R was significantly enhanced after 1/4 MIC tigecycline incubation. Otherwise, the question of whether biofilm formation with sub-MICs tigecycline is affected by the expression of other genes requires further investigation.

Nonaka et al. delineated that the mutation of nucleotides 965–967 in 16S rRNA is related to tetracycline resistance in *Helicobacter pylori* strains [65]. Nucleotide mutations can reduce the affinity of tetracycline to the target and result in resistance. We tested the nucleotide sequence of 16S rRNA and did not find point mutations. Therefore, an in-depth elucidation of other regulatory mechanisms is needed. With the availability of the complete genome sequence, transposon insertion has become a promising technique for screening drug-resistant genes [38]. In our study, 17978S was utilized as the recipient bacterium and the Tn5 transposon mutation library was effectively constructed with a capacity of 4500. After tigecycline resistance was derived with 1/4 MIC, we discovered that Tn5 has a great probability of randomly inserting into the *adeL* gene and inhibiting its expression, thereby affecting the tigecycline resistance of the strain. As mentioned above, the RND efflux system was responsible for multidrug resistance in *A. baumannii*. AdeL belongs to the LysR family of transcriptional regulators and was identified as negatively regulating *adeFGH*, which is also known as one of the RND efflux system-encoding genes overexpressed in antibiotic-resistant *A. baumannii* strains [66,67]. Gerson et al. also suggested amino acid mutations of Adel when tigecycline MICs increased [68]. Despite the research limitations on *adeL*, both this study and previous studies indicated that disturbance of *adeL* contributed to tigecycline resistance and provided a better understanding of the drug-resistant mechanisms in *A. baumannii*. We also found Tn5 was inserted into *ppc* and *recF*. *ppc* is involved in pyruvate metabolism [41] and *recF* is related to DNA replication/repair [42]. We did not find direct relationships between these two genes and antibiotic resistance. Further studies are needed to elucidate their functions.

## 4. Materials and Methods

### 4.1. Bacterial Strains and Antibiotic Susceptibility Monitoring

Three tigecycline-susceptible strains, A152S, A158S, and ATCC 17978 (named 17978S in this study), three tigecycline-intermediate clinical strains, A8S, A9S, and A11S, and one tigecycline-resistant strain, A54R, isolated from patients in the Second Affiliated Hospital of Nanchang University from 2011 to 2018 were used in this study. The tigecycline-susceptible strains were induced into tigecycline-resistant strains by sub-inhibitory concentrations (sub-MICs) of tigecycline and the tigecycline-resistant strain A54R was induced into a tigecycline-susceptible strain by high-temperature induction as detailed below.

Antibiotic susceptibility assays for the strains were performed in strict accordance with the Clinical and Laboratory Standards Institute (CISL) standard. Briefly, the strains were cultured overnight and inoculated into Mueller–Hinton (MH) liquid medium and the turbidities of the bacteria were adjusted to 0.5 McFarland (MCF) with MH broth after 4 h of culture. The antibiotic susceptibility to piperacillin (PIP), ceftazidime (CAZ), ceftriaxone (CRO), cefotaxime (CTX), cefepime (FEP), imipenem (IPN), piperacillin/tazobactam (PIT), gentamicin (GM), tobramycin (TM), minocycline (MNO), levofloxacin (LVF), and ciprofloxacin (CIP) was tested using the Kirby–Bauer method and susceptibility to polymyxin and tobramycin was tested by the agar dilution method. Susceptibility to tigecycline and ampicillin–sulbactam (ratio 2:1) was assessed using both the Kirby–Bauer method and the broth dilution method. For tetracycline, MICs of ≤2, 4, and 8 μg/mL were classified as susceptible, intermediate, and resistant, respectively.

The broth microdilution checkerboard method was used to determine whether the antibiotic had a synergistic effect. The bacterial solution was adjusted to 0.5 MCF; tigecycline and ampicillin–sulbactam (ratio 2:1) were diluted to 1/32~4 MIC according to the standard of 2012 CLSI. Then, 50 μL of tigecycline and 50 μL ampicillin–sulbactam dilutions were added to the wells in the column and rows, respectively, and then 100 μL bacterial solution was added to each well. The bacteria were cultured overnight and the MIC was analyzed. Fractional inhibitory concentration (FIC) calculation formula: FIC = FICA + FICB, FICA = MIC (antibiotic A and B combined)/MIC (antibiotic A mono-applied); FICB can be calculated similarly. Synergy: FIC ≤ 0.5, additive: 0.5 < FIC ≤ 1, irrelevant: 1 < FIC ≤ 4, antagonism: FIC > 4.

### 4.2. Induction of Tigecycline-Susceptible and Tigecycline-Intermediate Strains into Tigecycline-Resistant Strains

We used sub-MIC tigecycline to derive tigecycline-resistant strains from tigecycline-susceptible and tigecycline-intermediate strains [69]. Briefly, overnight cultured bacteria were inoculated at 1:20 into fresh LB broth containing 1/4 MIC of tigecycline and cultivated for 24 h, followed by passaging to the LB broth containing 1/2 MIC, 3/4 MIC, 1 MIC, and 3/2 MIC of tigecycline accordingly. If growth cessation appeared at a certain point, bacteria were cultivated at half of the tigecycline concentration for acclimatization. When the concentration approached 3/2 of the original MIC of tigecycline, the resulting MIC of the bacteria was evaluated with the broth dilution method. All tigecycline-susceptible or tigecycline-intermediate strains were induced in this manner until the MIC reached 64 μg/mL.

### 4.3. Induction of Tigecycline-Resistant Strain into Tigecycline-Susceptible Strain

A54R is a clinically isolated tigecycline-resistant strain that is routinely grown on LB plates with 2% agar at 37 °C. A single colony was picked and cultured in LB broth for 24 h at 42 °C. After subculturing for 18 generations (days) every 24 h, the MIC of tigecycline was appraised using the broth dilution method and those strains with MIC ≤ 2 μg/mL were defined as tigecycline-susceptible strain A54S.

### 4.4. Examination of Stability for Tigecycline Resistance

Single colonies of the induced tigecycline-resistant and tigecycline-susceptible strains were cultured in LB broth without antibiotics at 37 °C and sub-cultured in fresh broth every 24 h for 15 generations. Then, the MIC to tigecycline of the 15th generation was tested by the broth dilution method. The MIC before and after passage was compared to validate whether the strains had a stable tigecycline-resistance phenotype. At the same time, the induced strain A54S was cultured on LB solid medium without antibiotics or LB solid medium containing 8 μg/mL tigecycline and the number of colonies was compared to assess the recovery rate of tigecycline resistance.

### 4.5. Detection of the Fitness Cost of Tigecycline Resistance

The fitness cost of tigecycline resistance was represented by growth ability, motility, and bacterial competition ability. For growth ability, overnight cultures were passaged in LB broth or M9 basal medium (glucose content of 0.1%). Optical densities at 570 nm were monitored hourly over a 12 h period. The cell density of the 12 time points of a strain before and after induction was compared to measure the statistical significance. Bacterial motility was inspected on semisolid plates according to a previous description [70]. Briefly, bacterial suspensions were adjusted to 3 MCF and 2 μL was placed onto semisolid plates with 0.5% agar and incubated at 37 °C for 2 days. The diameters of the twitching zone were measured to compare differences in motility among strains. For cocultivation competition experiments, 17978 S/R (ratio 1:1) and A54 R/S (ratio 1:1) were cocultured at 37 °C overnight and then 10 μL was diluted to 10 mL fresh LB broth, which was further sub-cultured every 12 h until 96 h. The bacterial cocultures at 24, 48, 72, and 96 h were serially diluted and distributed on LB plates with or without 8 μg/mL tigecycline and, ultimately, the number of colonies was counted. All experiments were carried out in three biological replicates.

### 4.6. Determination of Biofilm Formation

Biofilm formation ability was detected based on the protocol of a previous study [62]. Overnight cultured bacteria were inoculated into M63 medium and harvested when MCF reached 0.5, which was then diluted at a ratio of 1:20. Afterwards, 200 μL of suspension was cultured in a 96-well plate at 37 °C for 24 h and further washed and incubated with 200 μL of 0.1% crystal violet at room temperature for 20 min. The crystal violet solution was discarded and 200 μL of 95% ethanol was added to dissolve the crystal violet. Absorbance of OD570 was detected by a microplate reader.

The biofilm formation abilities under the sub-MICs of tigecycline were also measured accordingly. Briefly, after detecting the MIC of each strain, bacterial suspensions were adjusted to 0.5 MCF. Then, the bacteria were incubated in MH liquid medium containing 1/8 MIC, 1/4 MIC, and 1/2 MIC concentrations of tigecycline at 37 °C for 24 h. The bacterial suspension without antibiotics was used as a positive control and then the biofilm was detected by crystal violet staining.

Furthermore, the MIC of the biofilm was determined by detecting the minimum biofilm eradication concentration (MBEC) [62]. First, the overnight cultured bacteria were diluted at a ratio of 1:100 into M63 medium and incubated in a shaker at 37 °C for 4 h. The turbidity of the bacterial suspension was adjusted to 0.5 MCF and diluted at a ratio of 1:20 with M63 medium. Then, 200 μL of bacterial suspension was cultivated in each well of a 96-well plate for 24 h before changing to fresh MH medium containing 0.125–2048 μg/mL tigecycline. After incubation at 37 °C for 24 h in the dark, the supernatant was discarded and washed three times with PBS. After incubation at 37 °C for another 24 h, the supernatant was discarded and washed three times with PBS. Finally, fresh cation-adjusted MH liquid medium without antibiotics was cultured at 37 °C for 18 h. The minimum antibiotic concentration that exhibited significant bacteriostasis was recognized as MBEC.

### 4.7. Investigation of Variability in 16S rRNA

According to the 16S rRNA sequence of 17978 in NCBI, two primers were designed (primers are listed in Table S2). The genomic DNA of each bacterium was extracted by a Bacterial Genomic DNA Extraction Kit (Solarbio, Beijing, China), followed by PCR amplification with high-fidelity Pfu DNA Polymerase (Promega, Beijing, China). The resulting fragments of each strain were sequenced and analyzed with BioEdit software 7.2 (https://bioedit.software.informer.com/7.2/) to detect variant sites.

### 4.8. Detection of Outer Membrane Integrity

Next, 5. -bromo-4-chloro-3-indolylphosphate (XP) staining was used to detect outer membrane integrity before and after induction [37]. Each overnight cultured bacterial strain was adjusted to 0.5 MCF and then 2 μL was pipetted onto an LB plate containing 40 μg/mL XP, which was further cultured at 37 °C for 24 h for observation. Meanwhile, the A54R strain treated with SDS served as a positive control and displayed blue precipitates in its colony.

### 4.9. Transposon-Insertion Sequencing

The plasmid pRL27, which carries the transposon Tn5 with a Km-resistance gene, was chosen as the vector. *A. baumannii* 17978S was chosen to be the original strain of the transposon mutation library. Overnight cultures of 17978S, *E. coli* CC118 λπ(pRL27), and *E. coli* DH5α (pRK2013) were cultured and then 100 μL of each bacterial suspension was mixed, centrifuged, and resuspended with100 μL LB, which was subsequently distributed on kanamycin-resistant LB plates and incubated overnight at 37 °C to screen for transposon-insertion mutants. Clones of the insertion mutants were picked up and cultured and stored as a transposon mutation library. To select for tigecycline-resistant mutants in the library, the mutants were incubated with 1/4 MIC of tigecycline for 24 h and then plated onto tigecycline-containing plates. If there was growth on the plate, the mutant was then selected for Tn5 insertion site identification with PCR and sequencing. Sequencing primers were designed using the transposon-insert sequence and the sequencing results were further compared with the 17978S genome to locate the inserted gene. The primers used in this study are listed in Supplementary Table S1.

### 4.10. Construction of the adeL Complementation Strain

To construct complementation strains carrying a plasmid expressing *adeL* in tigecycline-resistant strains, the *adeL* fragment was amplified and restriction digested and ligated to the pTrc99A plasmid. The successfully constructed plasmid was confirmed by both a PCR analysis and sequencing and then transferred into target strains by electric shock.

## 5. Conclusions

In conclusion, in this study, by reversing the susceptibility of tigecycline and comparing the initial strains and induced strains, we found that tigecycline resistance can cause fitness costs in the strains. Induction of tigecycline resistance can affect the susceptibility of strains to other antibiotics. Ampicillin–sulbactam may have potential use in the treatment of tigecycline-resistant bacteria. The mechanism of tigecycline resistance is complicated. We did not find 16S rRNA sequence mutations or cell membrane damage in this study. By constructing a Tn5 transposon mutation library, it was found that under the action of a sub-MIC of tigecycline, Tn5 has a great probability for randomly inserting into the *adeL* gene to improve the tigecycline resistance of the strain.

## Figures and Tables

**Table 1:** Antibiotic susceptibility of pre-induction and post-induction strains to 15 antibiotics.

Antibiotics	17978		A8		A9		A11		A152		A158		A54	
	S	R	S	R	S	R	S	R	S	R	S	R	R	S
PIP	R	I	R	R	R	R	R	R	R	R	R	R	R	R
CAZ	S	S	R	R	R	R	R	R	R	I	R	R	R	R
CRO	I	I	R	R	R	R	R	R	R	R	R	R	R	R
CTX	I	I	R	R	R	R	R	R	R	R	I	I	R	R
FEP	S	S	R	R	R	R	R	R	R	R	S	R	R	R
IPN	S	S	R	R	R	R	R	R	R	R	S	S	R	R
AMS	S	S	R	I	R	R	R	R	R	S	S	S	S	R
PIT	S	S	R	R	R	R	R	R	R	R	R	R	R	R
GM	I	I	R	R	R	R	R	R	R	S	I	R	R	R
TM	S	S	R	R	R	R	R	R	S	S	S	S	R	S
MNO	S	I	R	R	R	R	R	R	R	R	S	R	R	I
LVF	S	S	R	R	R	R	R	R	R	R	S	S	R	R
CIP	I	I	R	R	R	R	R	R	R	R	S	I	R	R
SXT	R	R	R	R	R	R	R	R	I	S	S	S	R	R
PMB	S	S	S	S	S	S	S	S	S	S	S	S	S	S

PIP: Piperacillin, CAZ: Ceftazidime, CRO: Ceftriaxone, CTX: Cefotaxime, FEP: Cefepime, IPN: Imipenem, AMS: Ampicillin/Sulbactam, PIT: Piperacillin/Tazobactam, GM: Gentamicin, TM: Tobramycin, MNO: Minocycline, LVF: Levofloxacin, CIP: Ciprofloxacin, SXT: Cotrimoxazole, PMB: Polymyxin. S: sensitive, I: intermediate, R: resistant.

**Table 2:** Antibiotic susceptibility of AMP, sulbactam, and AMS.

Strains	AMP (μg/mL)	Sulbactam (μg/mL)	AMS (2:1) (μg/mL)
17978S	32	2	2/1
17978R	32	2	2/1
A8S	>1024	1024	256/128
A8R	>1024	512	16/8
A9S	>1024	64	128/64
A9R	>1024	32	64/32
A11S	>1024	128	128/64
A11R	>1024	8	16/8
A152S	>1024	16	32/16
A152R	>1024	4	8/4
A158S	32	8	2/1
A158R	32	4	2/1
A54R	>1024	32	16/8
A54S	>1024	32	32/16

## Data Availability

Not applicable.

## Supplementary Materials

The following supporting information can be downloaded at: https://www.mdpi.com/article/10.3390/antibiotics11101379/s1. Table S1. The MICs to tigecycline of strains before and after induction and the tigecycline resistance stability of the induced strains. Table S2. Oligonucleotide primers used in this study. Figure S1. Growth curves of different strains of *Acinetobacter baumannii* before and after induction of tigecycline-resistance or susceptibility in LB broth. Figure S2. 16S rRNA sequence alignment.
