# Supplementary material for: Fitness Costs of Tigecycline Resistance in Acinetobacter baumannii and the Resistance Mechanism Revealed by a Transposon Mutation Library
Source: Antibiotics (Basel). 2022 Oct 9;11(10):1379. doi: 10.3390/antibiotics11101379 (PMC9598136; doi:10.3390/antibiotics11101379)

Table S1. The MICs to tigecycline of strains before and after induction and the tigecycline resistance stability of the induced strains.

Strains	MICs (μg/mL)	MICs after 15 successive sub-cultures (μg/mL)
A8S	4	-
A8R	128	128
A9S	4	-
A9R	64	64
A11S	4	-
A11R	64	64
A152S	2	-
A152R	128	128
A158S	0.5	-
A158R	>256	>256
17978S	0.5	-
17978R	128	128
A54R	8	-
A54S	1	1

-: means not tested.

Table S2. Oligonucleotide primers used in this study.

Primer	Sequence (5′-3′)
16S-P1	TTTAACTGAAGAGTTTGATCATGGC
16S-P2	TTCGTTAAGGAGGTGATCCAGCCGC
16S-P3	TAGCTGCGCCACTAAAGC
16S-P4	AGGAATACCGATGGCGAAGG
pRL-1	CGATGCGCCAGAGTTGTT
pRL-2	CTCACCGAGGCAGTTCCA
pRL-3	AACAAGCCAGGGATGTAACG
pRL-4	CAGCAACACCTTCTTCACGA
adeL-F	GTGGATCTATTTCATGCC
adeL-R	TTAAGTTTTGAGCGTATA
pTrc99A-F	ACTGCACGGTGCACCAATGC
pTrc99A-R	GCTCCCGGCGGATTTGTCCT

Figure S1. Growth curves of different strains of *Acinetobacter baumannii* before and after induction of tigecycline-resistance or susceptibility in LB broth. Data are presented as mean ± SD.

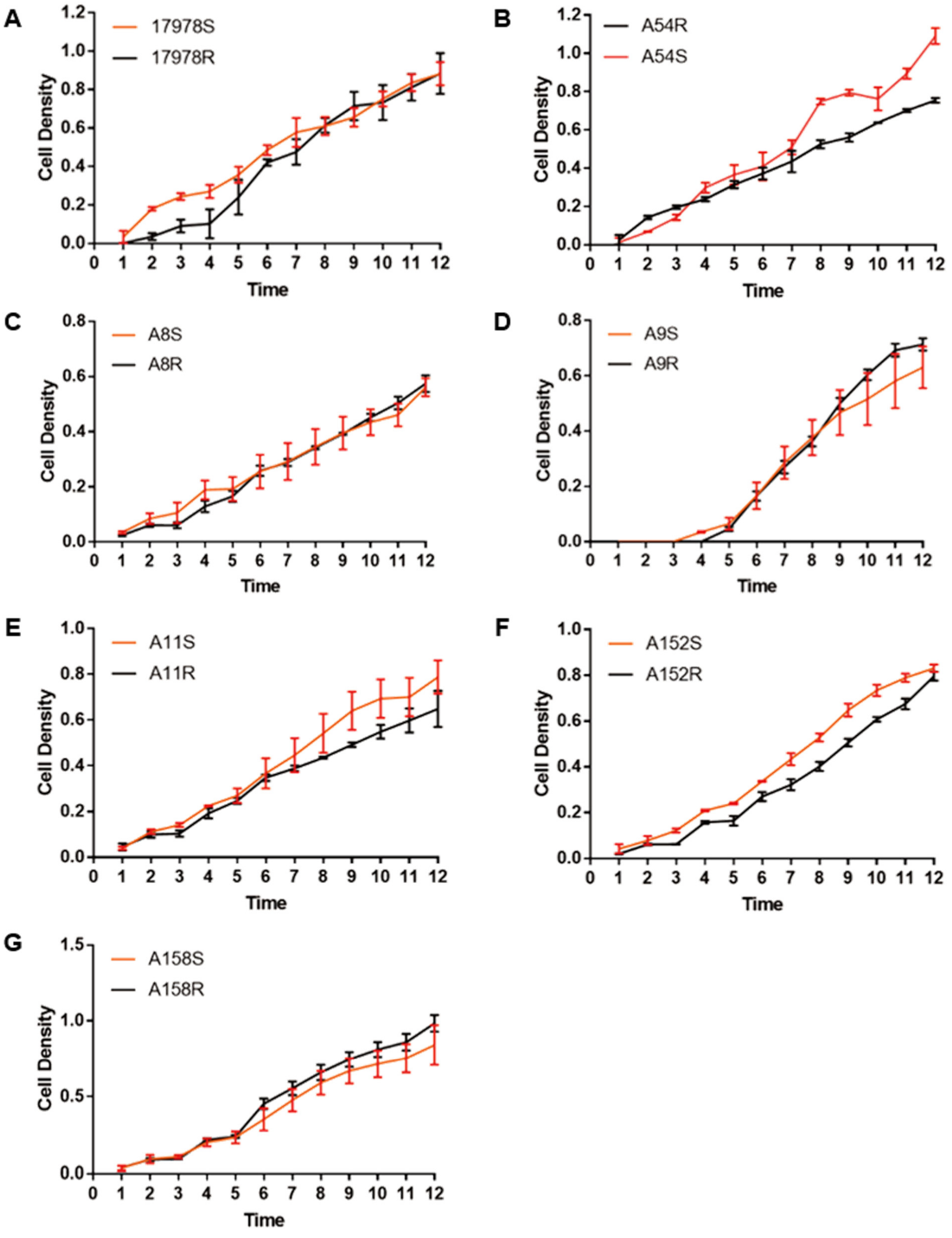

Figure S2. 16S rRNA sequence alignment.

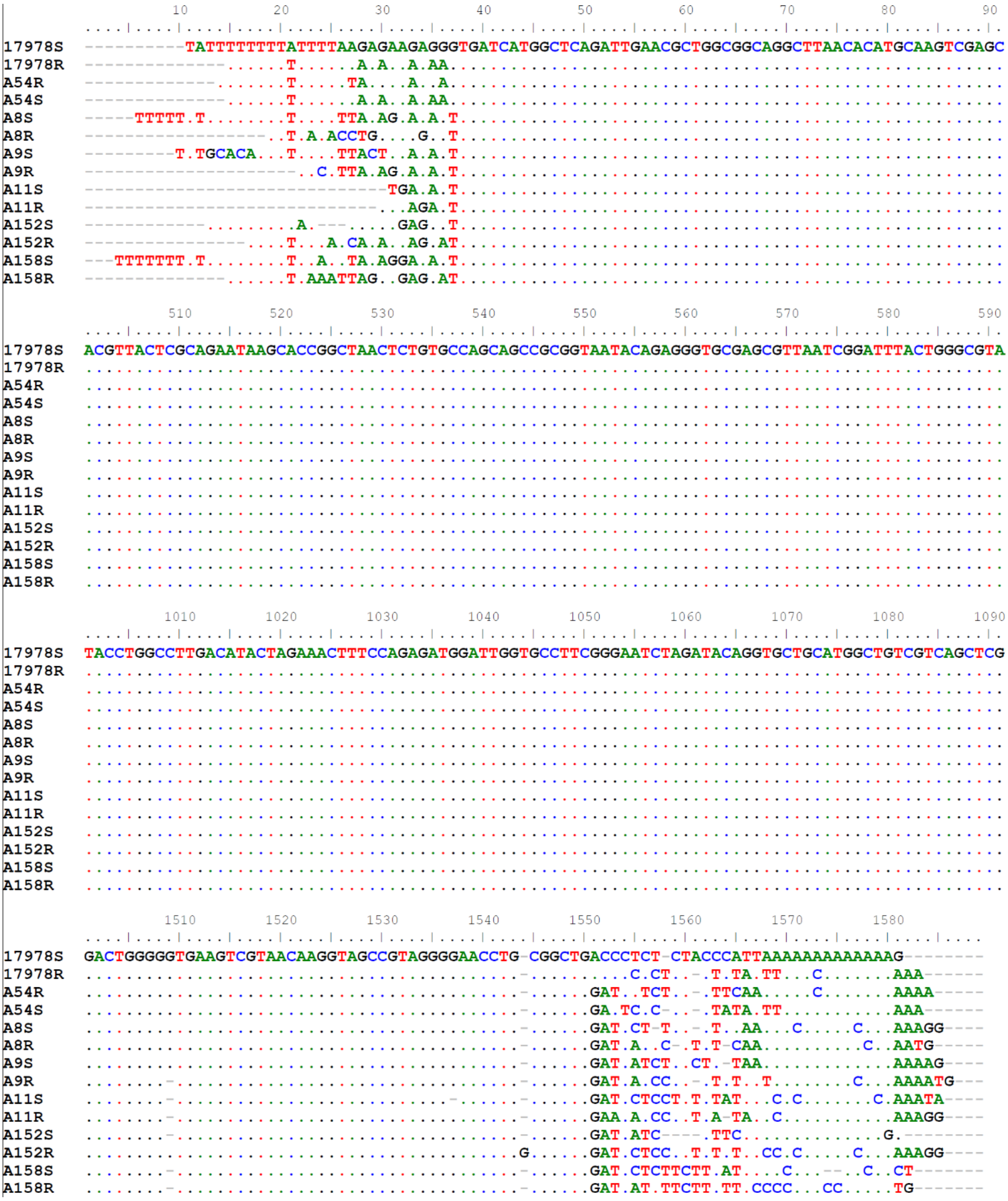

Supplement: Supplementary file 1 [file antibiotics-11-01379-s001.zip › antibiotics-1853064-supplementary.pdf]
